# Bloomingdale Asylum for the Insane

**Published:** 1832-02

**Authors:** 


### BLOOMINGDALE ASYLUM FOR THE INSANE.

*Cases of Puerperal Mania, &c.* By J. MACDONALD, M.D.†

B. G. ætat. nineteen. No hereditary predisposition to insanity, and never insane before; married almost two years; first child either stillborn, or died soon after birth; sanguine temperament: entered the Asylum, May 1st. Five weeks previously, after a natural labour, was delivered of a healthy child; for six days after confinement, "was doing well." At the expiration of this time, she became suddenly maniacal. Her treatment, adds the attending physician, was antiphlogistic, without relief.

Symptoms when admitted: General excitement; though unable to examine her pulse critically, we found it to be quick and full. Skin hot, as is common in this form of puerperal insanity; general disordered state of stomach and bowels; tongue coated in the centre, with red edges; bowels costive; no stool for several days; mind a perfect chaos, language obscene and incoherent; conduct furious; entire absence of moral propriety.

The first and most pressing indication was to remove the morbid condition of the primæ viæ. This was answered by repeated purging, with Sub. Mur. Hydr., both in large and small doses, Pulv. Purg., and Infus. Sen. comp.; and, when she refused these, Ol. Tiglii. During this course, however, typhoid symptoms manifested themselves, but subsided under its continuance. It may here be observed, that it is remarkable to what an extent it is necessary, in some cases of this kind, to pursue this method of treatment. Diet during this period, panada, gruel, arrowroot, light broths.

After the restoration of biliary and other secretions, the nervous excitement manifested by pervigilium, boisterous conduct, &c., next called the attention. From her aversion to medicine, our remedies were directed to the dermoid system alone. Tepid baths and frictions were used with the most tranquillising effect, inducing quietude and sleep, after days of commotion and nights of pervigilium. Counter-irritation was now produced by Ung. Ant. Tart. extensively applied to the hypochondria and extremities.

June 8th. The bowels having resumed their natural action, cathartic medicines are no longer exhibited. The baths proving still soothing, continue them. Being still highly excitable, it is necessary to confine her to her room, (except when exercising with the nurse,) entirely secluded from every thing calculated to call forth excitement. Mind still very incoherent, and, as is common in this form of puerperal insanity, conduct indelicate, and language exceedingly obscene.

July 1st. The digestive functions being now restored to a healthy condition, improvement in general health followed. This, however, being impaired by the heat of summer, a relapse of mental disorder ensued; inflammation of the mammæ, with symptomatic fever supervened. The subsidence of inflammation and fever, was succeeded by a tranquil mind; improvement in general health again took place, her mind daily and uninterruptedly gained strength, and she was discharged cured, October 5th, after five months' residence in the Asylum.

### II. *A Case of Puerperal Monomania.*

S. B. admitted into the B. A., July 15th, ætat. twenty-seven, married; temperament melancholic; has always been subject to headach at the catamenial period; gave birth to her second child four months since; and six weeks ago was observed to be palpably insane; ever since delivery, however, her conduct had been marked by many peculiarities, such as general torpor, loss of spirits, disinclination to mental and bodily exertion. She continued to nurse her child, until ten days previous to removal to this institution, when, in consequence of an attempt on his life, he was taken from her. She subsequently made several attempts at self-destruction. Before these occurrences, functional disorder was discovered in the mammæ, her milk had diminished in quantity, and had become watery. Two other circumstances point out the existence of some latent disorder long prior to the developement of insanity : viz. the entire absence of an erysipelatous inflammation, to which she had been long subject, particularly after her first confinement; and looseness of bowels, instead of habitual costiveness.

State when admitted: Entire absence of mental excitement, mind apparently correct upon every subject, except that of her recent conduct. This she speaks of in the most dispassionate manner; says that she is not, and has not been insane; that moral turpitude alone induced her to kill her child; that she deserves death; that she must be hung; that it was under such expectations she consented to come here; believing this to be the Penitentiary, where she was to await her trial, which would result in capital punishment. When told that her child was still alive and well, she replied, it was her intention to commit murder, and that she was equally as culpable as if he were dead. As soon as she found herself in the Asylum, she begged to be taken to the strongest cell in the house, chained to the floor, and treated in the harshest manner. Her bodily functions were not materially disturbed, but her eye bore the impress of a disordered mind; it was clouded, fixed and unobservant of external objects; its conjunctiva slightly tinged with yellow; tongue partially furred.

Management: She was partially indulged in her severe course of self-prescribed moral treatment, and with the happiest effects.

Her medical treatment was, a full emetic every third or fourth day, and counter-irritation by Ung. Ant. Tart. extensively applied to the surface. Her improvement was so considerable under this course, that her friends thought it expedient to remove her in about two weeks. Although her subsequent convalescence was less rapid, she ultimately recovered.

### III. *A Case of Puerperal Dementia.*

P. M. admitted into the B. A. 16th July; aged twenty-nine; married, and the mother of four children; temperament phlegmatic; became deranged for the first time immediately after parturition, and continued so for a year previous to being removed to this institution; of this period of insanity, there is no detailed account. At the time of admission, the more active stage had of course long since subsided, and had been succeeded by one of apathy, amounting to an almost entire cessation of mental action. From her general idiotic appearance, she was considered incurable, and, but for a gleam of intelligence that occasionally shone from her countenance, would have been deemed unworthy the trial of any curative means. The only impulse that gave rise to any degree of mental or physical exertion, was an attempt to give her medicine. Her husband and child, who now visited her, attracted no more attention than surrounding inanimate objects. She would sit or stand in the same posture for hours; her daily walk, if she took any at all, was confined to the gallery between her bedchamber and the dayroom. Towards all she observed the most stubborn taciturnity. Her countenance, from the long absence of passion and emotion, had assumed an inflexible, and for the most part vacant cast.

A most active course of moral, as well as medical treatment, was now adopted. She was obliged to make use of great muscular exertion, and was placed under the influence of various medicinal agents, but without radical change. The cloud would, indeed, at times seem to be driven from her mind, but was invariably succeeded by another, equally impenetrable. In this manner six months wore away, with an alternate dissipation and revival of hope. At this period, her thyroid gland was observed to be much enlarged. Supposing that her malady might possibly be connected with glandular obstruction, and judging from the effects of iodine in paralysis, it was thought advisable to commence a course of this medicine. Notwithstanding her extreme aversion to it, this remedy was faithfully exhibited, (though compulsion was necessary,) till its effects were evident in the glandular system, reducing the tumor in the neck, and restoring the menses, which had been suppressed from the onset of her insanity. Her mind now alternately gained and lost strength; its energies, however, seeming to increase with every successive effort. Every moral agent that would in the least tend to arouse her, was put in requisition. Besides the exercises of walking and riding, it was found useful to excite her in any way that would rouse her resentment.

The recovery of her affections kept pace with that of her mind, and was encouraged by a visit from her child. She now began to inquire into the nature of her situation, and asked after her husband and children. She soon wrote a letter to the former, who immediately visited and removed her. The journey home, and restoration to family, entirely reinstated her mind. Soon after arriving home, she addressed a letter to an individual connected with the Asylum, in which she says, "she felt like a person raised from the dead; that the two last years of her life had been a complete blank; of the transactions of which, she had not the slightest recollection." Her residence in the Asylum was just one year.

### IV. *A Case of Puerperal Mania.*

Mrs. S., ætat. twenty, of a sanguine temperament, a short time previous to admission in the B. A., (a few weeks at the furthest,) gave birth to her first child, immediately after which, we were informed in general terms, she became maniacal.

State when admitted: Countenance animated, and, when most so, flushed; at other times pale; eye exhibiting, in an unusual degree, the characteristic wildness and lustre of mania; nervous system to the greatest extent morbidly sensible; the opening of a door starts her from her seat, while the presence of a stranger seems to throw her into convulsive motions; manner playful and mischievous; mind in constant action; conversation incoherent and indelicate; pulse frequent; evidences of uterine irritation; tongue partially covered with a brown coat; bowels torpid. The lochial discharge flowing, though at first suppressed.

Treatment: Stomach freely evacuated by an emetic; followed by a cathartic course, and almost entire seclusion by confinement to her room. After remaining here nine days, was injudiciously removed home; but returned next day, her case so much aggravated, that severe personal restraint became necessary.

December 4th, (day after re-admission,) found her raving violently; language incoherent and obscene, constant delirium and pervigilium, tongue furred: was freely vomited without relief.

5th. ℞. Submur. Hydr. gr. xij., Pulv. Antimon. gr. xvj. M. et divid. in part. iv.; un. quaque 4ta hora.

6th. With an improved state of secretions, found her much more tranquil.

8th. Bowels torpid. Hab. Infus. Sen. comp., and continue its occasional use.

13th. Has been more tranquil for several days, but is still highly susceptible of external impressions; today much more excited; mind confused, face flushed, pulse accelerated, tongue pale. Hab. Pill. Gamb. comp. un. quaque hora ad operand.

16th. Cathartic operated copiously, without any alleviation of mental symptoms.

20th. Mind still variable and susceptible; begin counter-irritation, by the application of Ung. Ant. Tart.

January 2d. Insanity remitting in character; after lucid intervals, has paroxysms of frenzy during which she is entirely destitute of moral propriety. Hab. Solut. Antimon. gr. iij. cum Aqua ℥viij.

January 15th. For the last five or six days, has improved rapidly and uninterruptedly; her malady till this period having been remittent.

Under the constant use of the antimonial solution, which at the same time subdued vascular excitement and rendered the bowels sufficiently lax, an uninterrupted convalescence succeeded.

Discharged, recovered, January 31st, about ten weeks after admission.

### V. *A Case of Puerperal Dementia.*

A. Y., admitted 4th of August, with puerperal dementia, aged thirty; married; temperament phlegmatic. The only facts ascertained in relation to the history of her case were, that the attack was recent, that it succeeded parturition, and that her insanity, from its onset to the period of her admission, has observed the same character.

Condition when admitted: She is in a state of apathy, bordering on complete fatuity; sits for hours together in the same posture, without moving a muscle; is perfectly taciturn; is disinclined even to the exertion of taking food; and with this general prostration of mental and physical power, has lost every vestige of natural affection.

Her stomach, liver, and all organs connected with chylification, are in a corresponding state of torpor; skin sallow, conjunctiva yellow, breath oppressive, bowels costive, muscles placid.

Was immediately subjected to an active emetic, and cathartic course, with counter-irritation.

August 21st. Somewhat improved under this plan; eats more willingly, and speaks occasionally. Hab. baln. tepid. quaq. die tert. vel quart.

August 25th. No advantage gained by the use of the warm bath. Still manifests great disinclination to motion and mental exertion. Hab. baln. pluviale.

August 26th. Is considerably benefited by showering; answers when spoken to, and uses food more freely. Repeat shower bath twice a week, omitting, of course, the tepid bath, and using eccoprotics every second day.

September 9th. Under the above plan, she continues to improve; the functions of stomach and liver restored; menses, however, still suppressed.

Omit the above treatment. To restore the functions of the uterus, hab. Tinct. Senn. comp. ℥j. bis in die, et baln. tepid. quaque die 4ta.

Let her also exercise freely both by riding and walking. Diet nutritive. Under this course, the menses were re-established, and mind restored. Discharged, recovered, in October, between two and three months after admission. This was undoubtedly an idiopathic case of dementia, and not the result of previous excitement, since no such condition existed during any stage of the disorder.

Physicians, particularly those attached to public institutions, seldom witness the incipient stage of puerperal insanity; and this portion of the history of our cases, it is to be regretted, is necessarily either entirely omitted or very imperfectly delineated.

Among all the causes of insanity in females no single one perhaps is so frequent, as that arising from the puerperal period. Twenty-five cases of this kind have fallen under our observation at the Asylum, and in order to present them all with some of their more interesting circumstances, they are arranged in a synoptical table, in which Dr. M. gives a brief account of twenty-five cases of puerperal mania, from which he derives the following conclusions:

1. That young women are much more obnoxious to this malady than those more advanced, and that the susceptibility of females to it diminishes in about the same ratio that their years increase.

2. That women are more liable to this disease at the first, than at subsequent parturitions.

3. That a hereditary predisposition to insanity exists in less than one sixth of the cases.

4. That moral causes are co-operative only; and that, but in four cases out of twenty-five. As in part explanatory of this comparatively limited influence of moral causes, it may be observed, that all the patients were married.

5. That a large proportion of cases occurs within a few days after delivery; and that liability to this disease is found to diminish in proportion to the remoteness of the parturient period.

6. That puerperal insanity is one of the most curable forms of mental disorder.

7. That mania is the most common form of puerperal insanity;

8. And that this is far the most curable form.

9. That two thirds recover within six months, and that, in some rare cases, even when the disease is curable, it is protracted beyond two years.

10. That it seldom proves fatal.

